# Sex Modifies Metabolic Pathways Associated with Lipids in Untargeted Metabolomics: The Coronary Artery Risk Development in Young Adults (CARDIA) Study, 2005–2006

**DOI:** 10.3390/metabo15110730

**Published:** 2025-11-08

**Authors:** Autumn G. Hullings, Annie Green Howard, Katie A. Meyer, Christy L. Avery, Kari E. North, Sachin Mhatre, Wei Sha, Yuanyuan Li, Blake R. Rushing, Susan Sumner, Xiuxia Du, Cora E. Lewis, Penny Gordon-Larsen

**Affiliations:** 1 Department of Nutrition, Gillings School of Global Public Health, University of North Carolina at Chapel Hill, Chapel Hill, NC 27599, USA; ktmeyer@email.unc.edu (K.A.M.); blake_rushing@unc.edu (B.R.R.); susan_sumner@unc.edu (S.S.); pglarsen@unc.edu (P.G.-L.); 2 Carolina Population Center, University of North Carolina at Chapel Hill, Chapel Hill, NC 27516, USA; aghoward@email.unc.edu (A.G.H.); christy_avery@unc.edu (C.L.A.); 3 Department of Biostatistics, Gillings School of Global Public Health, University of North Carolina at Chapel Hill, Chapel Hill, NC 27599, USA; 4 Nutrition Research Institute, University of North Carolina at Chapel Hill, Chapel Hill, NC 28081, USA; yuan0451@hotmail.com; 5 Department of Epidemiology, Gillings School of Global Public Health, University of North Carolina at Chapel Hill, Chapel Hill, NC 27599, USA; kari_north@unc.edu; 6 Department of Biostatistics and Data Sciences, Levine Cancer Institute, Atrium Health, Charlotte, NC 28204, USA; smhatre@unc.edu (S.M.); wei.sha@atriumhealth.org (W.S.); 7 Department of Bioinformatics & Genomics, University of North Carolina at Charlotte, Charlotte, NC 28223, USA; xiuxia.du@charlotte.edu; 8 Department of Epidemiology, School of Public Health, University of Alabama at Birmingham, Birmingham, AL 35294, USA; celewis@uabmc.edu

**Keywords:** sex-modification, lipids, lipoproteins, untargeted metabolomics, blood plasma

## Abstract

**Background**: There are differences in lipid metabolism by sex that are relevant for health, but metabolic pathways are not fully understood. We investigated sex differences in cross-sectional associations between metabolic pathways identified using untargeted metabolomics and clinical lipid measures (total cholesterol [TC], triglycerides [TG], and low- and high-density lipoprotein cholesterol [LDL-c; HDL-c]) from blood plasma in the Coronary Artery Risk Development in Young Adults (CARDIA) study (Year 20; 2005–2006). Our objective was to determine whether associations between metabolic pathways and lipid measures differ by sex and to identify pathways that may underlie sex-specific mechanisms of lipid metabolism. **Methods**: Using data from 2169 participants, (44% women, mean age = 45, 58% White, 42% Black), we used: (1) Orthogonal partial least squares-regression (OPLS-R) to compare variation in TC, TG, LDL-c, and HDL-c explained by metabolites in men vs. women, (2) linear regression to assess sex-modification of associations between 7255 metabolite peaks and lipid measures using false discovery rate (FDR)-corrected *p* < 0.1, and (3) pathway enrichment analyses to identify metabolic pathways that differed by sex using Fisher’s exact test (FET) *p* < 0.05. **Results**: We found that: (1) untargeted metabolomic data reflected variation in lipid measures better for men compared to women, (2) associations between metabolite peaks and lipid measures differed by sex, and (3) 8 unique pathways differed by sex, particularly primary bile acid biosynthesis, linoleic acid metabolism, and arginine biosynthesis. **Conclusions**: Our findings suggest distinct lipid-associated metabolic activity by sex that points to potential mechanistic pathways.

## 1. Introduction

Cardiovascular diseases (CVD) are among the leading drivers of morbidity and mortality in the United States [1,2]. Clinical lipid measures, including total cholesterol (TC), triglycerides (TG), low-density lipoprotein cholesterol (LDL-c) and high-density lipoprotein cholesterol (HDL-c), are well established risk factors for CVD [3]. Higher circulating levels of TC, TG, and LDL-c, and lower levels of HDL-c are associated with greater atherosclerotic plaque burden, hypertension, inflammation, and heart failure [4]. Sex differences in lipid measures are well-documented [5,6,7], but underlying mechanisms remain poorly understood.

Understanding sex differences in these mechanisms is important given the underdiagnosis and undertreatment of CVDs, particularly among women, who have historically been understudied in CVD-related research and are recognized as an important risk subpopulation [8,9]. Biological sex, defined by genetic, hormonal, and physiological characteristics, influences lipid measures and CVD [6,10,11,12,13,14,15,16,17]. While higher levels of TC, TG, and LDL-c and lower levels of HDL-c are atherogenic in men and women, differences in lipoprotein profiles by sex could indicate sex-specific variations in metabolism [18,19,20]. For example, pre-menopausal women may have higher lipoprotein lipase activity and fatty acid utilization, potentially altering metabolic pathways that affect lipoprotein levels [6,19]. Additionally, post-pubertal women have nearly twice the concentration of large HDL-c particles compared to men [9,21]. Estrogen’s influence on lipid metabolism may partially explain these differences, but it remains unclear whether sex differences in lipid levels reflect distinct underlying metabolic pathways in men and women.

Untargeted metabolomics is a technique that captures thousands of metabolites—many of which are unidentified—from biological samples and can uncover metabolic pathways underlying sex differences in lipid levels [22]. Although sex differences in metabolism [6,23] and lipids [19,24] have been described, prior studies were limited by small sample sizes, lack of diversity, or insufficient power to detect differences by sex. To address these gaps, we investigated whether sex modifies associations between metabolite peaks from untargeted metabolomics and clinical lipid measures. We hypothesized that associations metabolite-lipid associations differ by sex, potentially revealing sex-specific metabolic pathways relevant for CVD reduction and prevention.

## 2. Materials and Methods

### 2.1. Study Population

The CARDIA study is an ongoing, prospective cohort that examines the development of CVD risk factors in participants of self-reported Black and White race [25]. Recruitment of CARDIA participants was approximately balanced by sex, race, education (≤high school and >high school), and age (18–24 and 25–30 years) in Birmingham, AL, Chicago, IL, Minneapolis, MN, and Oakland, CA. In total, 5115 participants were recruited at baseline (1985–1986). By 2005–2006, 72% of the surviving participants attended the year 20 follow-up examination [26,27]. Our cross-sectional analysis included year 20 participants with a fasted blood sample for whom metabolomics analyses were completed (n = 3391). All exclusions are listed in Supplemental Figure S1. Our final analytic sample included 2169 participants, including 964 women and 1205 men.

### 2.2. Plasma Sample Preparation and Untargeted Metabolomics Analysis

All participants in our analytic sample completed blood plasma sample collection and metabolomic profiling (Supplemental Methods). Untargeted metabolomics were conducted using Vanquish ultra-high-performance liquid chromatography (UHPLC-MS) coupled with Q ExactiveTM HF-X Hybrid Quadruple-OrbitrapTM Mass Spectrometer (Thermo Fisher Scientific, San Jose, CA, USA). Missing peak data were imputed using random forest [28,29]. Data were median-scaled and log2 transformed across each metabolite to improve normality for linear regression models. Pareto scaling was used for Orthogonal Partial Least Squares–Regression (OPLS-R). We identified/annotated 7522 metabolite peaks as per published protocols [30,31,32,33], which were used in linear regression and multivariate models.

### 2.3. Clinical Lipid Measures

TC, TG, LDL-c, and HDL-c are primary outcomes of interest due to known differences by sex and clinical significance. Other lipid measures, such as apolipoprotein B and non-HDL-c, were not included because they are highly correlated with LDL-c and not currently recommended in clinical lipid panels [3,34,35]. Lipids were measured at year 20 from fasted (≥12 h) blood plasma samples [36]. We added a constant to all lipid measures (TC: +52.1, TG: +18.4, LDL-c: +49.9, HDL-c: −2.3), to account for statin use [37,38,39].

### 2.4. Biological Sex and Covariates

Biological sex in CARDIA was self-reported. Covariates included study sample metabolomics data acquisition batch number (42-level categorical), study field center (Birmingham, AL; Chicago, IL; Minneapolis, MN; Oakland, CA), age (continuous), self-reported race (Black/White), highest attained education (≥12 years/<12 years), smoking status (current/former/never), validated physical activity intensity score (continuous; theoretical range 0–2184 exercise units, higher scores represented greater physical activity) [40], alcohol consumption (continuous; mL/day), hypertension status (yes/no) defined as systolic blood pressure ≥ 140 mmHg or diastolic blood pressure ≥ 90 mmHg or on anti-hypertensive medication, diabetes status (yes/no) defined as elevated fasting glucose levels ≥ 126 mg/dL or 2 h glucose tolerance test ≥ 200 mg/dL or hemoglobin HbA1c ≥ 6.5% or on diabetic medications but not pregnant, total energy intake (continuous, kcals) [41], estimated glomerular filtration rate (eGFR) [42], currently taking birth control medication (women only; yes/no), menopausal status by menstrual cycle (pre-menopausal: regular cycle, peri-menopausal: irregular cycle in the last 12 months, post-menopausal > 12 months since last cycle), and body mass index (BMI; continuous kg/m^2^). All covariates were chosen based on evidence of associations with lipid measures [43,44,45].

### 2.5. Statistical Analyses

Our statistical analyses methods have been published previously [33]. Our primary objective was to investigate sex modification of associations between circulating metabolites with TC, TG, LDL-c, and HDL-c. We used three main analytic strategies to achieve this objective, each with complementary strengths and goals: (1) heterogeneity by sex of model performance in predicting each lipid measure using all 7255 metabolite peaks with OPLS-R multivariate analysis, (2) multivariable-adjusted linear regression models of the association between all 7522 metabolite peaks with interactions between each peak and sex, (3) metabolomic pathway enrichment analyses to discover metabolic pathways with differential enrichment between sexes. We conducted all analyses using SAS version 9.4 (SAS Institute Inc., Cary, NC, USA) for multivariable-adjusted regression models, SIMCA software (version 17) for the multivariate analyses, and Mummichog algorithm (version 2.0) within the MetaboAnalyst version 5.0 software (https://www.metaboanalyst.ca/) for pathway enrichment analyses.

#### 2.5.1. Orthogonal Partial Least Squares–Regression (OPLS-R)

We used OPLS-R to evaluate metabolite patterning and model performance using all 7522 metabolite peaks with each lipid measure by sex since OPLS-R cannot evaluate interactions. OPLS-R uses supervised learning to comprehensively assess the predictive ability (Q2) “cross-validated R2 value” and accuracy (normalized root mean squared error [nRMSE]) of multivariate data (i.e., untargeted metabolomic data) to explain variation in a continuous variable (i.e., each lipid measure), with higher Q2 values indicating better predictive ability [46]. To assess the statistical significance of the Q2 value, we performed permutation-based validation tests using 999 permutation datasets. We randomly selected 70% of our sample as the training set and the remaining 30% as the test set. We used cross-validation within the training dataset to generate models and then used the test dataset to evaluate model performance against an independent dataset (Supplemental Table S1). The training set was used to generate sex-specific metabolite scores (linear combinations of peaks), and the test set was used to evaluate the prediction accuracy of these scores. We used the normalized root mean squared error (nRMSE) to assess model prediction accuracy, calculated by dividing the rMSE by the difference in the range (maximum–minimum) [47]. We compared the nRMSE from the metabolite scores calculated in each sex separately to evaluate whether variation in lipid measures was better explained in men or women, with lower nRMSE indicating better predicting accuracy. While the split-sample design was necessary for testing model performance in OPLS-R, all regression and pathway analyses used the full dataset to maximize statistical power.

#### 2.5.2. Unstratified Linear Regression Models with Sex-Metabolite Peak Interactions

To assess whether sex modified associations between metabolite pathways and lipids, we used multivariable-adjusted linear regression models with sex-metabolite peak interactions (PROC GLM, SAS 9.4, SAS Institute Inc., Cary, NC, USA). We ran regression models separately for each of the 7522 metabolite peaks—defined by their mass-to-charge ratio and retention time—and the 4 clinical lipid measures, separately, with adjustments for multiple comparisons using false discovery rates (FDR) < 0.1. An FDR threshold between 5 and 10% is commonly used in -omics literature [48,49,50]. We used an FDR < 0.1 as a conservative approach to ensure adequate statistical power to detect effect modification. Because this untargeted metabolomics dataset contains mostly unannotated peaks, these models were used to identify pathways with statistically different metabolic activity by sex rather than to interpret individual metabolites. We included an interaction term between sex (reference = women) and each metabolite peak to test whether associations with lipid measures differed by sex. We used a stepwise modeling approach to evaluate the robustness of associations; we included models adjusted for basic demographics, lifestyle, and clinical covariates, separately, as listed in Table 1. Our fully adjusted model included metabolomics data acquisition batch number, study field center, age, self-reported race, highest level of education achieved, total energy intake, BMI, smoking status, physical activity score, alcohol consumption, taking birth control medications (women only), diabetes status, hypertension status, and eGFR. While the fully adjusted model provides the most comprehensive adjustment, less complex models allowed us to isolate the potential influence of grouped covariates—non-modifiable demographics, lifestyle behaviors, and clinical factors—on the observed associations.

#### 2.5.3. Unstratified Pathway Enrichment

Given the incomplete identification of metabolite peaks from untargeted metabolomics, we used pathway enrichment analyses to derive biologically interpretable results and identify metabolic pathways with differential metabolic activity by sex. We used metabolite features (i.e., mass-to-charge ratio [*m*/*z*]) and raw *p*-values from the sex-metabolite interaction term from linear regression models to conduct pathway analyses using the Mummichog algorithm (version 2) within the MS Peaks to Pathways Functional Analyses module within MetaboAnalyst (version 6). We also conducted pathway enrichment analyses from OPLS-R results for pathways with variable importance projection (VIP) scores > 1.5 [54] using an Enrichment Factor (ratio of the number of significant metabolite peaks observed vs. expected in a pathway based on Fisher’s exact test (FET) ≤ 0.05) by sex. Compounds with greater metabolic activity than expected by chance were considered “enriched”. We ran similar models based on raw *p*-values from the sex-stratified models, without interaction terms, to identify pathways associated with lipid measures in men and women. To determine metabolic enrichment, we mapped all 7522 metabolite peaks onto their respective human Kyoto Encyclopedia of Genes and Genomes (KEGG) pathways (database release 102.0). All analyses were conducted in positive ion mode with a mass tolerance set at 3 ppm.

#### 2.5.4. Sensitivity Analyses

We conducted several sensitivity analyses. Given known differences in lipid measures by menopausal status in women, we first assessed potential differences across self-reported pre-menopausal, peri-menopausal, or post-menopausal status [55,56]. We ran the fully adjusted models (excluding the sex-interaction term) with pathway enrichment analysis (as described above). We considered only those metabolic pathways that differed significantly by sex in unstratified, fully adjusted linear regression models with sex-metabolite interactions. Second, given the unequal distribution of participants on lipid-lowering medications and the potential of bias, we ran a sensitivity analysis with adjustment for lipid-lowering medication use (yes/no) rather than the constants applied in the main analysis. We ran unstratified, fully adjusted linear regression models and compared pathway results with vs. without constants. Finally, we ran unstratified, fully adjusted linear regression models excluding metabolite peaks with >80% missing and compared pathway results.

### 2.6. Ethical Considerations and Approvals

The Institutional Review Board at each CARDIA site approved the exam protocols. The metabolomics ancillary study was approved at UNC. The CARDIA study participants gave their consent at each follow-up exam. The CARDIA Steering Committee approved the parent study (R01DK139598). We utilized the STROBE cross-sectional checklist when writing our manuscript [57].

## 3. Results

### 3.1. Participant Characters

Our analytic sample characteristics are shown in Table 1, which demonstrates covariates and clinical lipid measures by sex. Overall, the mean (sd) age was 45 (3.7), 44% of participants were women, and 58% reported White race. Compared to women, men had significantly higher TC, TG, and LDL-c, but lower HDL-c (*p* < 0.05; Table 1).

### 3.2. Comparison of Metabolomic Data Patterning and Model Performance by Sex Using Orthogonal Partial Least Squares–Regression (OPLS-R)

**Figure 1:**
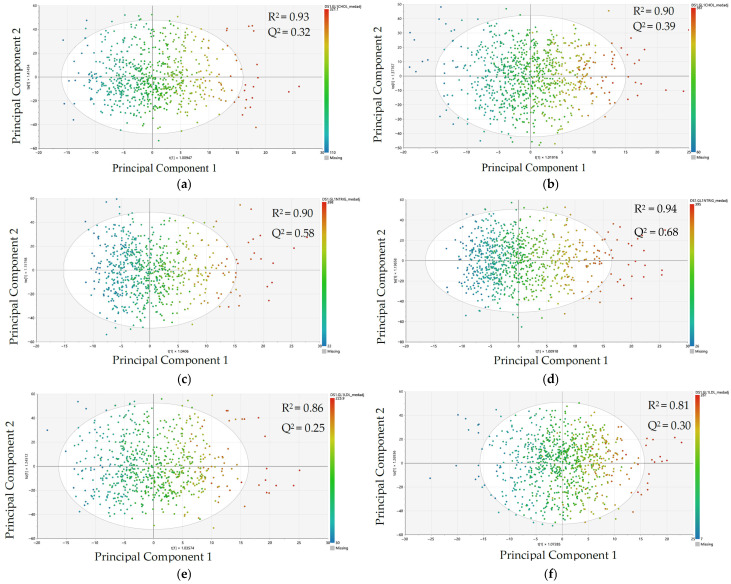

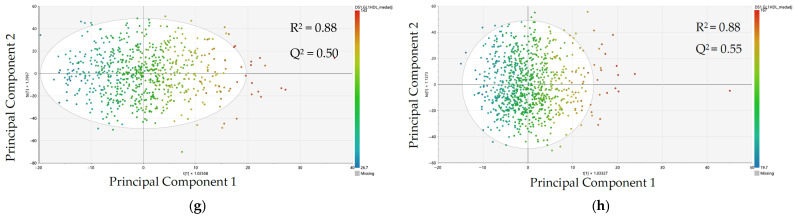
Orthogonal partial least squares scatter plots generated from training data (70% of full sample) for associations between principal components (linear combinations of metabolite peaks) and each clinical lipid measure in the Coronary Artery Risk Development in Young Adults Study, 2005–2006 (N = 2169) for (**a**) total cholesterol in women, (**b**) total cholesterol in men, (**c**) triglycerides in women, (**d**) triglycerides in men, (**e**) LDL-c in women, (**f**) LDL-c in men, (**g**) HDL-c in women, and (**h**) HDL-c in men. Each point represents a participant.

We used sex-stratified OPLS-R models to evaluate metabolomic patterning and model performance of the ability of metabolite peak data to predict TC, TG, LDL-c, and HDL-c (Figure 1). To evaluate model prediction, Figure 1 presents scatter plots generated from training data (70% of the sample) showing associations between principal components (linear combinations of metabolite peaks) and each clinical lipid measure for women and men. Principal component 1 (the predictive component) explains variation in each lipid measure, while principal component 2 (the orthogonal component) explains variation in the metabolite peaks unrelated to the lipid measure. Each point represents a participant (darker red = higher lipid levels; darker blue = lower lipid levels).

For men, we observed adequate predictive ability (Q2 > 0.40) for TC, TG, and HDL-c, but not LDL-c. For women, we observed adequate predictive ability (Q2 > 0.40) for TG and HDL-c, but not TC or LDL-c. However, Q2 values were higher than expected by chance (*p* < 0.001) for each sex, indicating that metabolites can significantly discriminate higher and lower values of clinical lipid measures for both sexes. We also assessed the accuracy of the metabolite peak data to explain each clinical lipid measure. Using test data, we observed similar nRMSE values by sex, but prediction accuracy was consistently higher for men compared women, as indicated by the lower nRMSE value, across all lipid measures except for triglycerides (Supplemental Figure S2). Adjusting the models for covariates improved the prediction for men and women, but patterns remained the same. (Supplemental Table S2).

### 3.3. Sex Modification of Metabolite Peak-Lipid Associations in Unstratified Linear Regression Models (7255 Metabolite Peaks and 4 Clinical Lipid Measures)

Of the total 7522 metabolite peaks, sex modified associations in fully adjusted models (sex-metabolite interaction raw *p* < 0.05) for 405, 939, 671, 1098 metabolites peaks for TC, TG, LDL-c, and HDL-c, respectively (Supplemental Table S3). After adjusting for multiple comparisons using an FDR < 0.1, 85 (1%) and 83 (1%) for TG and HDL-c remained significant, respectively. We did not observe any significant sex-metabolite interactions for TC or LDL-c using an FDR < 0.1.

### 3.4. Sex Differences in Metabolic Pathway Activity Associated with Clinical Lipid Measures Using Pathway Enrichment Analyses

**Figure 2:**
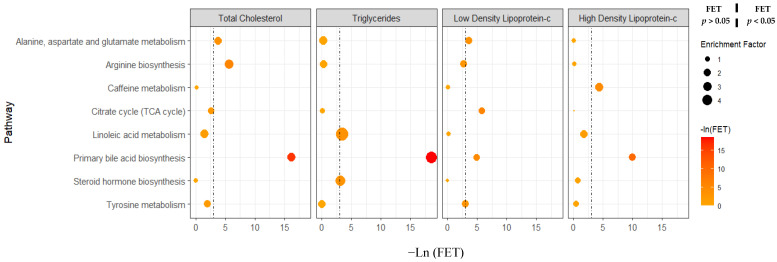
Unstratified fully adjusted models with sex-metabolite interactions comparing metabolic pathways with significant (FET < 0.05) differential activity by sex for at least one clinical lipid measure in the CARDIA study, 2005–2006 using the full dataset. Models adjusted for analytic batch, field center, total energy, age, self-reported race, highest education achieved, smoking status, physical activity score, body mass index, diabetes status, hypertension status, and estimated glomerular filtration rate.

From fully adjusted models with sex-metabolite peak interactions, we identified 56, 58, 64, and 59 total pathways associated with TC, TG, LDL-c, and HDL-c, respectively (Supplemental Table S4). Across the 4 clinical lipid measures, we identified 8 unique pathways that differed by sex (FET < 0.05) for at least one measure (Figure 2) including lipid metabolism (primary bile acid biosynthesis, steroid hormone biosynthesis, and linoleic acid metabolism), carbohydrate metabolism (citrate/TCA cycle), protein metabolism (alanine, aspartate and glutamate metabolism, arginine biosynthesis, and tyrosine metabolism) and caffeine metabolism. In Figure 2, differential activity was determined by Pathway Enrichment Analysis (Mummichog algorithm v2, MetaboAnalyst v5) with human KEGG pathway mapping [58].FET *p* < 0.05 indicates pathways with greater metabolic activity in the pathway than expected by chance with darker red colors indicate greater significance [58]. The Enrichment Factor represents the ratio of the number of metabolites found vs. expected in a pathway with higher ratios (larger circles) indicating greater pathway activity than expected by chance [58]. The statistical significance depended on the lipid measure. Only primary bile acid biosynthesis differed significantly by sex for all clinical lipid measures (all FET < 0.01). Of note, alanine, aspartate and glutamate metabolism differed significantly by sex for TC and LDL-c (both FET < 0.05), but the remaining 6 pathways differed for only one clinical lipid measure; arginine biosynthesis for TC (FET < 0.01), steroid hormone biosynthesis and linoleic acid metabolism for TG (both FET < 0.05), citrate/TCA cycle and tyrosine metabolism for LDL-c (both FET < 0.05), and caffeine metabolism for HDL-c (FET < 0.05).

From the additional basic demographic-, lifestyle-, and clinical-adjusted models, we identified 8, 11, and 8 unique pathways, respectively, that differed by sex (FET < 0.05) for at least one clinical lipid measure (Supplemental Table S4). Across all models, 3 pathways were consistently different by sex including arginine biosynthesis for TC, linoleic acid metabolism for TG, and primary bile acid biosynthesis for all 4 lipid measures. Across all models except the basic demographic model, alanine, aspartate and glutamate metabolism differed by sex TC and LDL-c and the citrate (TCA) cycle for LDL-c only. Steroid hormone biosynthesis and tyrosine metabolism were consistently different by sex for TG and LDL-c, respectively, for all models except the clinical model. For the basic demographic model, drug metabolism, other enzymes were significantly different by sex for TC only, lysine degradation for LDL-c only, and steroid biosynthesis for TG and HDL-c (Supplemental Figure S3). For the lifestyle model, alanine, aspartate, and glutamate metabolism significantly differed by sex for TC and LDL-c and the citrate (TCA) cycle for LDL-c, as seen in the fully adjusted model (Supplemental Figure S4). Finally, results from the clinical model were most like the fully adjusted model except for steroid hormone biosynthesis and tyrosine metabolism, which were not statistically significant (Supplemental Figure S5).

### 3.5. Sensitivity Analyses

**Figure 3:**
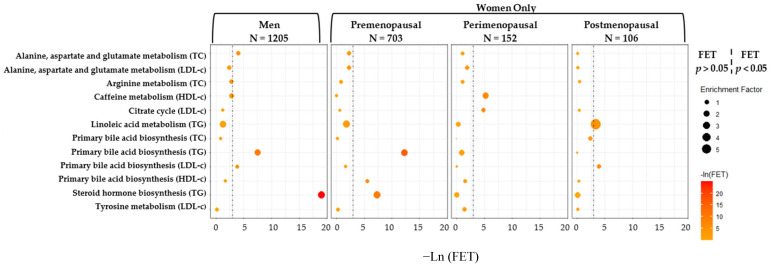
Sex stratified model results of metabolic pathways in men and women (by menopausal status) from fully adjusted models (excluding sex-metabolite interactions) that were significantly associated (FET < 0.05) with at least one clinical lipid measure by sex in the CARDIA study, 2005–2006 using the full dataset.

In addition to the interaction models, we report results from sex-specific sensitivity analyses in men (n = 1205) and in women by menopausal status (pre-menopausal [n = 703], peri-menopausal [n = 152], and post-menopausal [n = 106]) in Supplemental Table S5. Overall, we observed more differences for peri- and post-menopausal women compared to men in linear regression models with pathway enrichment (Figure 3). For example, primary bile acid biosynthesis remained statistically significant for TG but in men and premenopausal women only. Additionally, sensitivity results from sex-stratified OPLS-R pathway enrichment models (Supplemental Tables S6 and S7) revealed similar significant pathways (VIP score > 1.5 and FET < 0.05) as in the linear regression models, but with differing patterns of significance. For example, primary bile acid biosynthesis remained significant for LDL-c and TG in men, but only HDL-c in women. From our second sensitivity analyses using lipid medication-adjusted models without constants, 7 of the 8 unique metabolic pathways remained statistically significant, except for caffeine metabolism for HDL-c only and steroid hormone biosynthesis for triglycerides only (Supplemental Table S4). In addition, ubiquinone and other terpenoid-quinone biosynthesis became statistically significant for HDL-c only (FET = 0.03). In our third sensitivity analysis, which excluded metabolite peaks with >80% missingness, we observed the same significant pathways as our main results, except that biosynthesis of unsaturated fatty acids was additionally significant for triglycerides and HDL-c (Supplemental Table S8).

## 4. Discussion

Several metabolic pathways have been linked to CVD and dyslipidemia [53], including medium- and long-chain acylcarnitines [59], short-chain fatty acids (e.g., butyrate) [60], polyunsaturated fatty acids (e.g., linoleic acid) [61,62], phospholipid-related metabolites (e.g., trimethylamine-N-oxide) [63,64,65,66], amino acids (e.g., phenylalanine, glutamate) [67,68], sphingolipids [69,70,71], and ceramides [71,72,73]. However, few studies have investigated sex differences in associations between circulating metabolites and clinical lipid measures using untargeted metabolomics data.

Our primary objective was to investigate whether associations between untargeted metabolomics profiles and clinical lipid measures varied by sex using data from 2169 participants in the CARDIA study. Using all 7522 metabolite peaks identified from untargeted metabolomics, our results support the hypothesis that associations between a select number of metabolic pathways and clinical lipid measures differ by sex. Overall, we found that: (1) metabolomic patterning and model performance differed by sex, with untargeted metabolomic data explaining more variation in lipid measures and with better model performance in men compared to women; (2) associations between metabolite peaks and clinical lipid measures differed by sex for a limited number of peaks; and (3) across the four clinical lipid measures, eight distinct pathways differed by sex, with primary bile acid biosynthesis, linoleic acid metabolism, and arginine biosynthesis having the most prominent differential activity.

We used OPLS-R models to evaluate sex-specific differences in overall metabolomic patterning and whether comprehensive metabolite profiles explained lipid variation differently by sex. We observed adequate model performance (Q^2^ > 0.40) for TC, TG, and HDL-c in men, and for TG and HDL-c in women, with Q^2^ values exceeding what would be expected by chance (*p* < 0.001) for all models. Model fit, measured by nRMSE, was also generally stronger for men than women across most lipid measures. To gain biological insight, we conducted pathway enrichment using metabolite peaks with VIP scores > 1.5 from each model. These features represent the most influential contributors, and their pathway-level aggregation allowed us to identify enriched biological processes, many of which differed by sex. Together, these findings suggest that associations between metabolomic profiles and lipid levels may differ by sex, with stronger model performance observed in men. This supported our rationale for conducting regression models and pathway enrichment analyses with sex-metabolite peak interactions to further explore potential sex-specific metabolic pathways related to lipid metabolism.

Given the inherent limitations of untargeted metabolomics datasets—including incomplete metabolite annotation, difficulty in reliably identifying individual metabolites, and greater measurement variability at the individual-metabolite level—we prioritized pathway-level analyses over individual metabolite results to ensure robustness and biological interpretability. Pathway enrichment methods allowed us to detect changes in metabolic pathway activity that reflect shared biochemical processes, providing mechanistic insights despite incomplete metabolite identification.

The strongest and most consistent sex differences in metabolic pathways were related to lipid metabolism. We observed statistically significant sex interactions for primary bile acid biosynthesis across all clinical lipid measures (FET < 0.001). Our results corroborate well-established differences in bile acid pool composition and bile acid synthesis by sex [23,74,75,76]. Bile acids function as metabolic regulators that are important in oxidative stress mediation, cholesterol regulation, and atherosclerotic plaque formation [77]. Several key enzymes involved in bile acid synthesis, including certain aldo-keto reductase and cytochrome P450 enzymes, and are also regulated by sex hormones [75]. Thus, bile acid synthesis modulation is a promising target for sex-specific strategies to reduce CVD. Currently, statins are commonly prescribed to treat CVD, which reduce LDL concentrations, cholesterol biosynthesis, and atherosclerotic plaque formation [3,78]. Although the efficacy of statins is similar in men and women [79], women are more likely to experience negative side effects and discontinue treatment compared to men [78]. Given persistent underdiagnosis and undertreatment of CVD in women [8,9], identifying sex differences in bile acid metabolism may be an important first step to highlight alternative treatment options for women, which may be more effective in cases where statins are less tolerated [75].

We also observed significant sex differences in the associations between linoleic (omega-6 polyunsaturated fatty acid [PUFA]) metabolism with TG. Linoleic acid is an essential omega-6 fatty acid commonly found in Western diets. Replacing saturated fatty acids with PUFAs lowers circulating TG and LDL-c and improves cardiovascular health in men and women [80,81,82,83]. However, differences in sex hormones may impact PUFA metabolism, specifically in the conversion of essential omega-3 and omega-6 PUFAs into their longer-chain metabolites [84,85,86,87]. Compared to men, women tend to have better dietary habits and healthier CVD profiles [88,89]. However, intervention studies have demonstrated that even when consuming the same diet, men and women metabolize diet differently, particularly for PUFA metabolism, which may explain the distinct associations by sex in plasma lipid measures [90,91].

We also found sex differences in the associations of protein (alanine, aspartate and glutamate metabolism, arginine biosynthesis, and tyrosine metabolism), carbohydrate (citrate/TCA cycle), and caffeine metabolism. Although not specifically related to lipid measures, others have found sex differences in phenylalanine (an alanine derivative) and glutamate [92,93,94], tyrosine [93,94,95,96,97], citrate/TCA cycle [98,99], and caffeine metabolism [100]. Reasons for sex differences in associations between non-lipid related pathways and lipid measures are less well characterized, though evidence suggests factors upstream of metabolism, including sex differences in genetics [24,101,102,103] and sex-specific variations in microbiota [76,104,105], as areas for further research.

Although limited by sample size, we included a sensitivity analysis stratified by sex in men and in women by menopausal status [55,56]. Our results suggest that lipid-associated metabolism varies by menopausal status, which aligns with previous findings [96,106]. Patterns of enrichment and significance appeared most similar between men and pre-menopausal women, while lipid-associated metabolic activity was most statistically significant among perimenopausal women. However, given the small sample size and self-reported menopausal status, such comparisons should be interpreted with caution. While we were unable to determine the direction of associations, our findings provide insight into important pathways that may differ by sex and menopausal status and point to metabolic pathways for targeted analyses and further investigation.

As our study was a cross-sectional analysis, we were unable to determine causal relationships or rule out the possibility of residual confounding. We did not account for genetic or microbiome data, which have sex-specific associations with lipid measures [24,76,103]. While our study focused on standard lipid measures (TC, TG, LDL-c, HDL-c), the absence of non-HDL-c and apo-B is a limitation, as these markers may provide more nuanced insights into sex-specific differences in metabolite-lipid associations. Furthermore, data for this analysis were collected during the CARDIA Year 20 exam (2005–2006), which is the most recent time point with concurrent diet and untargeted metabolomics data available. While dietary habits and the food environment may have changed over the past two decades, this dataset provides a valuable benchmark for identifying metabolic pathways relevant to early cardiometabolic disease prevention and reflects an era prior to widespread consumption of ultra-processed foods. CARDIA remains one of the few large, population-based cohorts with detailed dietary data during midlife—a critical window for assessing early cardiometabolic risk. Our analyses used random forest imputation to address missing data, which performs well in untargeted metabolomics where missingness is often a mix of random (e.g., due to alignment issues) and informative (e.g., low-abundance metabolites) mechanisms [28,29]. Despite these limitations, we assessed the robustness of our findings in a series of models adjusting for demographic, lifestyle, and clinical confounders. We recommend further validation in other populations—Future research leveraging newer metabolomic datasets as they become available in CARDIA will be important for advancing women’s health and understanding metabolic changes across the menopausal transition.

## 5. Conclusions

Our results are consistent with the hypothesis that lipid-associated metabolic activity is modified by sex for at least some pathways. Identifying sex differences in pathways related to clinical lipid measures is promising for pointing to mechanistic candidates that could be targets for lifestyle and drug interventions to mitigate CVD, particularly given sex differences in diagnosis and treatment [8,9].

## Figures and Tables

**Table 1:** Participant characteristics by sex in the Coronary Artery Risk Development in Young Adults Study, 2005–2006 (N = 2169).

Variable	Women (n = 964)		Men (n = 1205)	
Clinical lipid measures ^1^				
Total cholesterol (mg/dL), mean (sd) *	188.1	(34.1)	191.7	(37.4)
Triglycerides (mg/dL), mean (sd) *	90.7	(50.2)	121.3	(67.0)
Low-density lipoprotein cholesterol (mg/dL), mean (sd) *	109.8	(31.2)	119.8	(34.4)
High-density lipoprotein cholesterol (mg/dL), mean (sd) *	60.1	(16.7)	47.5	(14.2)
Basic demographics ^2^				
Study field center, n (col %) *				
Birmingham, AL	222	(23.0)	334	(27.7)
Chicago, IL	241	(25.0)	290	(24.1)
Minneapolis, MN	213	(22.1)	306	(25.4)
Oakland, CA	288	(29.9)	275	(22.8)
Self-reported race, n (col %)				
White	544	(56.4)	714	(59.3)
Black	420	(43.6)	491	(40.7)
Education, n (col %) *				
High school or less	108	(11.2)	208	(17.3)
College or more	856	(88.8)	997	(82.7)
Age (years), mean (sd) *	44.7	(3.8)	45.3	(3.5)
Total energy intake (kcals), median (25th, 75th) *	1842.5	(1447.0, 2401.1)	2522.6	(1964.0, 3259.6)
Lifestyle factors ^3^				
Smoking status, n (col %) *				
Never	593	(61.5)	758	(62.9)
Former	220	(22.8)	209	(17.3)
Current	151	(15.7)	238	(19.8)
Taking birth control medication				
No	834	(86.5)	-	-
Yes	130	(13.5)	-	-
Physical activity score ^4^, median (25th, 75th) *	234	(106.5, 436.5)	360	(200.0, 588.0)
Alcohol consumption (mL/day), mean (sd) *	8.2	(14.5)	14.5	(25.9)
Body mass index and clinical factors ^5^				
Diabetes status ^6^, n (col %) *				
No	896	(92.9)	1087	(90.2)
Yes	68	(7.1)	118	(9.8)
Hypertension status ^7^, n (col %) *				
No	745	(77.3)	864	(71.7)
Yes	219	(22.7)	341	(28.3)
Estimated glomerular filtration rate (mL/min/1.73 m^2^), mean (sd)	95.6	(14.3)	94.8	(15.2)
Body mass index (kg/m^2^), mean (sd)	28.9	(7.5)	28.9	(5.7)
Taking lipid-lowering medications, n (col %) *				
No	912	(94.6)	1049	(87.1)
Yes	52	(5.4)	156	(12.9)
Sensitivity analysis				
Self-reported menopausal status, n (column %) ^8^				
Pre-menopausal	703	(73.2)	-	-
Peri-menopausal	152	(15.8)	-	-
Post-menopausal	106	(11.0)	-	-

* Significantly different by sex based on *p*-value < 0.05 using the full dataset. ^1^ Constants were added to all lipid measures to account for statin use (TC: +52.1, TG: +18.4, LDL-c: +49.9, HDL-c: −2.3). ^2^ The basic demographics model adjusted for analytic batch, study field center, age, self-reported race, highest education achieved, and total energy intake, with sex*metabolite peak interaction term. ^3^ The lifestyle factors model adjusted for basic model covariates + smoking status, and physical activity score, with sex*metabolite peak interaction term. ^4^ Physical activity score ranged from 0 to 2184 (higher scores represented greater physical activity) [33,40]. ^5^ The clinical factors model adjusted for basic model covariates + estimate glomerular filtration rate, diabetes status, body mass index, and hypertension status, with sex × etabolite peak interaction term. ^6^ Diabetes status: having elevated fasting glucose ≥ 126 mg/dL or 2 h glucose tolerance test ≥ 200 mg/dL or hemoglobin HbA1c ≥ 6.5% or on diabetic medications but not pregnant [33,51] ^7^ Hypertension status: having SBP ≥ 140 mmHg or DBP ≥ 90 mmHg or on hypertensive medications [33,52]. ^8^ Self-reported menopausal status was defined among women who had not had a hysterectomy as having a regular menstrual cycle in the past 12 months (pre-menopausal), having an irregular menstrual cycle in the past 12 months (peri-menopausal), or >12 months since last menstrual cycle [53].

## Data Availability

The data from the findings of this study are available upon request.

## Supplementary Materials

The following supporting information can be downloaded at: https://www.mdpi.com/article/10.3390/metabo15110730/s1, Figure S1: Study Flowchart; Table S1: Participant characteristics by sex in training (70% of data) and test (30% of data) datasets in the Coronary Artery Risk Development in Young Adults Study, 2005–2006 (N = 2169); Table S2: Fit statistics and model predictive ability in sex-stratified samples for each clinical lipid measure using test data (30% of full dataset); Figure S2: Predicted vs. observed ability of the 7522 metabolite peaks to predict each clinical lipid measure using the test data (30% of full dataset); Table S3: a–d: Unstratified model results for metabolic pathways differentially associated with lipid measures by sex in the Coronary Artery Risk Development in Young Adults Study, 2005–2006 (N = 2169) using the full dataset; Figure S3: Unstratified model 1 (basic demographic model) a results for statistically significant metabolic pathways (FET < 0.05) differentially associated with at least one clinical lipid measure by sex in the Coronary Artery Risk Development in Young Adults Study, 2005–2006 using the full dataset; Figure S4: Unstratified model 2 (lifestyle-adjusted model) a results of significant metabolic pathways (FET < 0.05) differentially associated with at least one clinical lipid measure by sex in the Coronary Artery Risk Development in Young Adults Study, 2005–2006 using the full dataset; Figure S5: Unstratified model 3 (clinical-adjusted model) a results of significant metabolic pathways (FET < 0.05) differentially associated with at least one clinical lipid measure by sex in the Coronary Artery Risk Development in Young Adults Study, 2005–2006 using the full dataset; Table S4: a–d: Stratified model results in men and women (by menopausal status) for metabolic pathways significant associated (FET < 0.05) with at least one clinical lipid measure by sex in the Coronary Artery Risk Development in Young Adults Study, 2005–2006 using the full dataset; Table S5: a–d: Unstratified and stratified OPLS-R metabolic pathway enrichment results of pathways significantly associated (VIP score > 1.5, FET < 0.05) with at least one clinical lipid measure in the Coronary Artery Risk Development in Young Adults Study, 2005–2006 using the full dataset; Table S6: Unstratified fully adjusted linear regression results of metabolite peak features associated with each clinical lipid measure in the Coronary Artery Risk Development in Young Adults Study, 2005–2006; Table S7: a–d: Unstratified and stratified OPLS-R results of metabolite peak features and VIP scores associated with each clinical lipid measure in the Coronary Artery Risk Development in Young Adults Study, 2005–2006; Table S8: Metabolic pathways identified from 7488 metabolite peaks (excluding metabolites peaks with >80% missingness) associated with each clinical lipid measure with differential metabolic activity by sex in the Coronary Artery Risk Development in Young Adults Study, 2005–2006 (N = 2169); Figure S6: Principal components analysis of all study samples, study pools, NIST reference samples, and blanks using all 7522 metabolomics features used in the Coronary Artery Risk Development in Young Adults Study, 2005–2006; Supplemental Methods.

## Abbreviations

The following abbreviations are used in this manuscript:

ADAP	Automated Data Analysis Pipeline
BMI	Body mass index
CARDIA	Coronary Artery Risk Development in Young Adults
CVD	Cardiovascular diseases
FDR	False discovery rate
FET	Fisher’s exact test
HDL-c	High-density lipoprotein cholesterol
IPSL	In-house physical standard library
LDL-c	Low-density lipoprotein cholesterol
MS	Mass spectrometry
M/Z	Mass-to-charge ratio
nRMSE	Normalized root mean square error
nRMSEP	Normalized root mean square error of prediction
OPLS-R	Orthogonal partial least squares—regression
PUFA	Polyunsaturated fatty acid
RT	Retention time
SD	Standard deviation
TC	Total cholesterol
TG	Triglycerides
UHPLC-MS	Ultra-high performance liquid chromatography–high-resolution mass spectrometry
VIP	Variable Influence on Projection
