# Supplementary material for: Sex Modifies Metabolic Pathways Associated with Lipids in Untargeted Metabolomics: The Coronary Artery Risk Development in Young Adults (CARDIA) Study, 2005–2006
Source: Metabolites. 2025 Nov 8;15(11):730. doi: 10.3390/metabo15110730 (PMC12654688; doi:10.3390/metabo15110730)
Supplement: Supplementary file 1 [file metabolites-15-00730-s001.zip › Supplemental Tables_Figures_Methods.pdf]

SUPPLEMENTAL MATERIAL

Supplemental Figure S1. Study Flowchart*

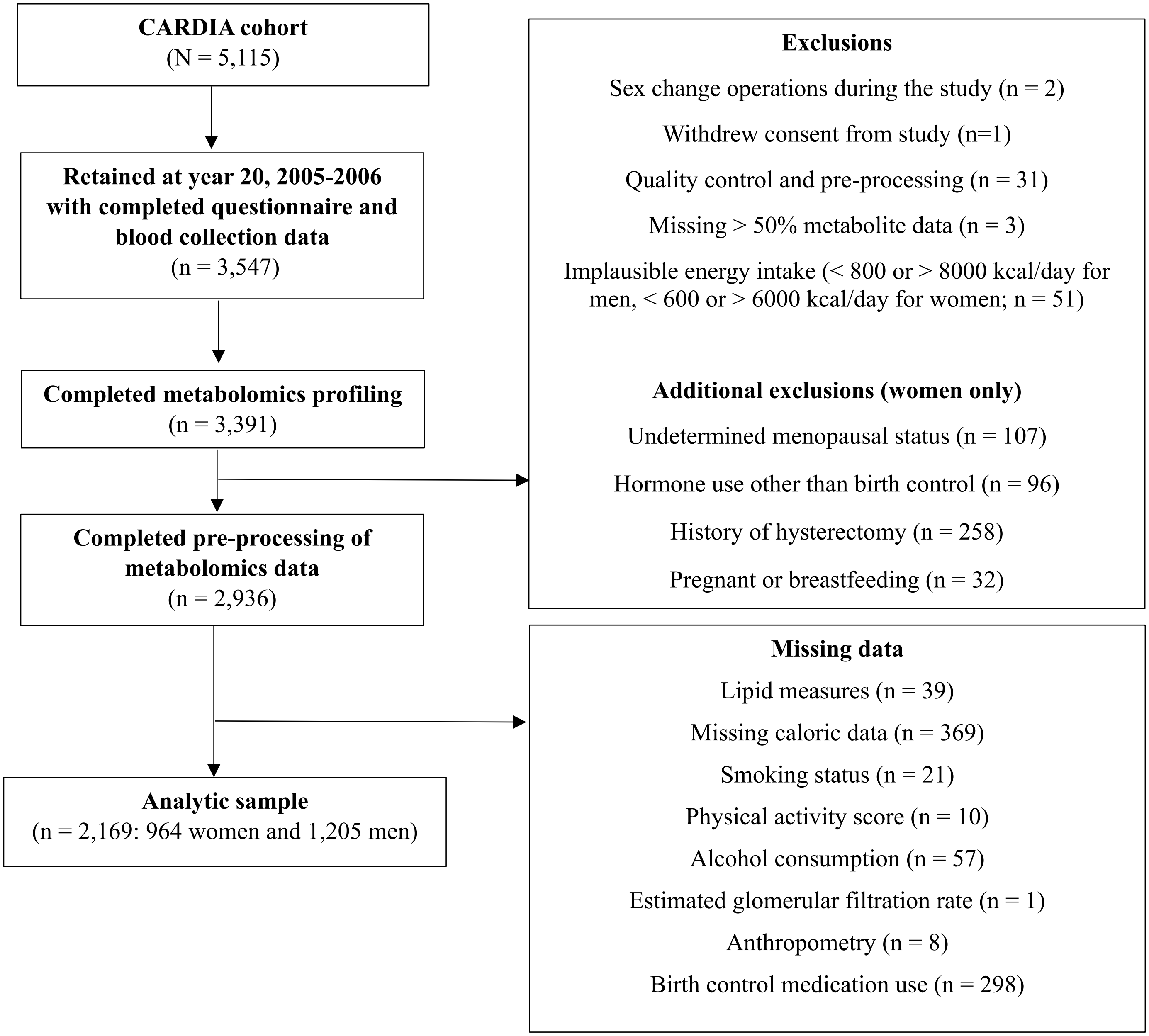

*Some participants had more than one exclusion.

Supplemental Table S1. Participant characteristics by sex in training (70% of data) and test (30% of data) datasets in the Coronary Artery Risk Development in Young Adults Study, 2005–2006 (N = 2,169)

Variable	Test dataset				Training dataset			
	Women (n = 277)		Men (n = 352)		Women (n = 687)		Men (n = 853)	
**Age (years), mean (sd)**	44.32	(3.77)	45.12	(3.60)	44.89	(3.76)	45.36	(3.44)
**Body mass index (kg/m^2), mean (sd)**	28.56	(6.75)	29.09	(5.86)	29.08	(7.82)	28.85	(5.61)
**Total energy intake (kcals), median (25th, 75th)**	1949.53	(726.50)	2787.64	(1223.78)	2020.47	(812.05)	2728.7	(1122.90)
**Physical activity score [a], median (25th, 75th)**	273.57	(251.63)	421.21	(309.26)	310.01	(257.58)	415.7	(288.84)
**Alcohol consumption, mean (sd)**	8.21	(14.63)	15.06	(28.12)	8.14	(14.38)	14.30	24.90
**Glomerular filtration rate (ml/min/1.73 m^2), mean (sd)**	95.74	(14.01)	95.18	(15.36)	95.52	(14.46)	94.60	(15.17)
**Study field center, n (col %)**								
**Birmingham, AL**	72	(25.99)	99	(28.13)	150	(21.83)	235	(27.55)
**Chicago, IL**	72	(25.99)	84	(23.86)	169	(24.60)	206	(24.15)
**Minneapolis, MN**	56	(20.22)	84	(23.86)	157	(22.85)	222	(26.03)
**Oakland, CA**	77	(27.80)	85	(24.15)	211	(30.71)	190	(22.27)
**Self-reported race, n (col %)**								
**White**	157	(56.68)	202	(57.39)	387	(56.33)	512	(60.02)
**Black**	120	(43.32)	150	(42.61)	300	(43.67)	341	(39.98)
**Education, n (col %)**								
**High school or less**	33	(11.91)	63	(17.90)	75	(10.92)	145	(17.00)
**College or more**	244	(88.09)	289	(82.10)	612	(89.08)	708	(83.00)
**Smoking status, n (col %)**								
**Never**	174	(62.82)	222	(63.07)	419	(60.99)	536	(62.84)
**Former**	60	(21.66)	51	(14.49)	160	(23.29)	158	(18.52)
**Current**	43	(15.52)	79	(22.44)	108	(15.72)	159	(18.64)
**Diabetes status [b], n (col %)**								
**No**	258	(93.14)	314	(89.20)	638	(92.87)	773	(90.62)
**Yes**	19	(6.86)	38	(10.80)	49	(7.13)	80	(9.38)
**Hypertension status [c], n (col %)**								
**No**	207	(72.92)	262	(74.43)	538	(78.31)	602	(70.57)
**Yes**	70	(25.27)	90	(25.57)	149	(21.69)	251	(29.43)

[a] Physical activity score ranged from 0 to 2184 (higher scores represented greater physical activity).[34,41]
[b] Diabetes status: having elevated fasting glucose ≥126 mg/dL or 2-hour glucose tolerance test ≥200 mg/dL or hemoglobin HbA1c ≥ 6.5% or on diabetic medications but not pregnant [34,105]
[c] Hypertension status: having a systolic blood pressure ≥140 mmHg or diastolic blood pressure ≥90 mmHg or on hypertensive medications. [34,106]

Supplemental Table S2. Fit statistics and model predictive ability in sex-stratified samples for each clinical lipid measure using test data

	Women					Men				
**Clinical lipid measure [a]**	**% variance explained [b]**	**rMSE [c]**	**nRMSE [d]**	**Q^2**	**R^2**	**% variance explained**	**rMSE**	**nRMSE**	**Q^2**	**R^2**
**Total cholesterol**										
**Model 1 [e]**	40.15%	29.70	0.12	0.32	0.93	45.54%	26.39	0.11	0.40	0.90
**Model 2 [f]**	54.34%	25.90	0.10	0.32	0.94	55.65%	23.82	0.10	0.40	0.90
**Triglycerides**										
**Model 1**	67.27%	33.65	0.08	0.57	0.90	68.31%	37.77	0.10	0.68	0.94
**Model 2**	78.38%	26.48	0.07	0.58	0.91	73.65%	34.62	0.09	0.68	0.94
**Low density lipoprotein - cholesterol**										
**Model 1**	31.33%	28.09	0.14	0.25	0.86	42.25%	26.27	0.11	0.30	0.81
**Model 2**	46.49%	24.66	0.13	0.25	0.87	52.43%	23.77	0.10	0.31	0.83
**High density lipoprotein - cholesterol**										
**Model 1**	53.25%	11.69	0.12	0.50	0.88	55.58%	10.59	0.08	0.55	0.88
**Model 2**	65.29%	9.98	0.10	0.50	0.89	67.41%	9.01	0.07	0.55	0.90

[a] Constants were added to all lipid measures to account for statin use (TC: +52.1, TG: +18.4, LDL-c: +49.9, HDL-c: -2.3)
[b] Percent (%) variance explained by each model
[c] Root mean square error (rMSE): lower RMSE indicates better model fit
[d] Normalized root mean squared error (nRMSE) was calculated by dividing the rMSE by the difference of the sex-specific range (maximum – minimum) of each clinical lipid measure; lower nRMSE indicated higher model accuracy
[e] Model 1 (derived in sex-stratified samples): Clinical lipid measure = $\beta_0 + \beta_1$(first principal component of the metabolite score) + ε
[f] Model 2 (derived in sex-stratified samples): Clinical lipid measure = $\beta_0 + \beta_1$(first principal component of the metabolite score) + β_2(Batch) + β_3(Field Center) + β_4(Total Energy) + β_5(Age) + β_6(Race) + β_7(Education) + β_8(Smoking Status) + β_9(Physical Activity) + β_{10}(Alcohol Consumption) + β_{11}(Birth Control Use-women only) + β_{12}(BMI) + β_{13}(Diabetes) + β_{14}(Hypertension) + β_{15}(eGFR) + ε

Supplemental Figure S2. Predicted vs. observed ability of the 7,522 metabolite peaks to predict each clinical lipid measure using the test data

a) Total cholesterol

Women

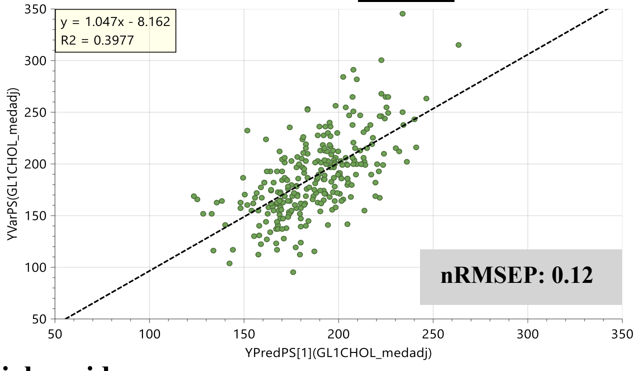

Men

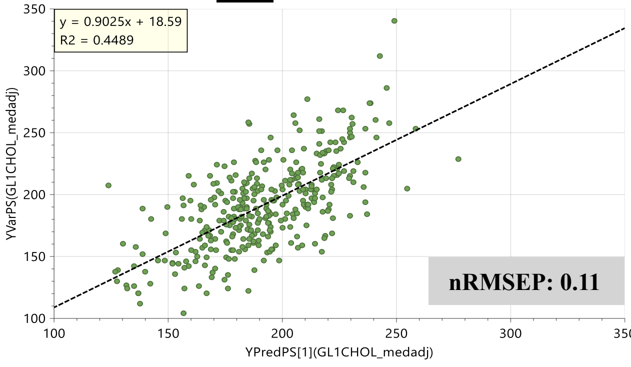

b) Triglycerides

Women

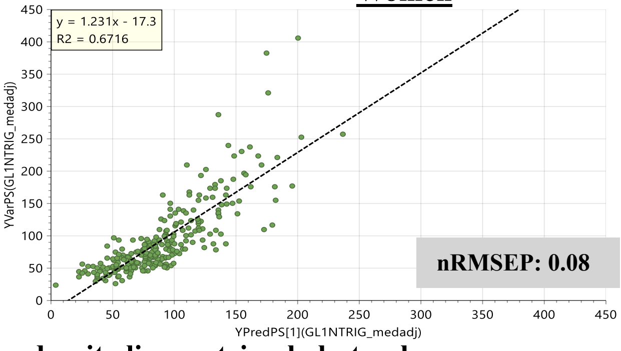

Men

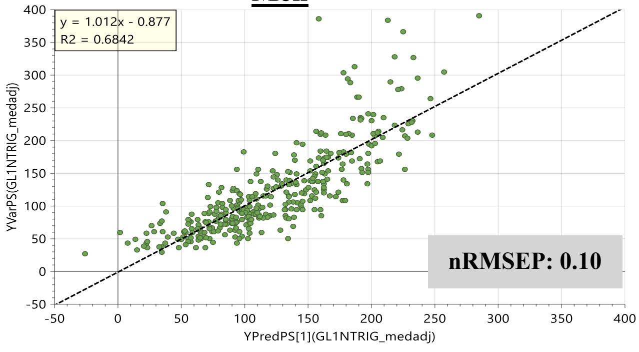

c) Low density lipoprotein-cholesterol

Women

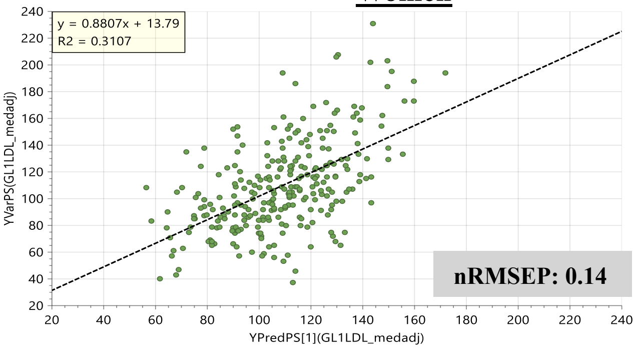

Men

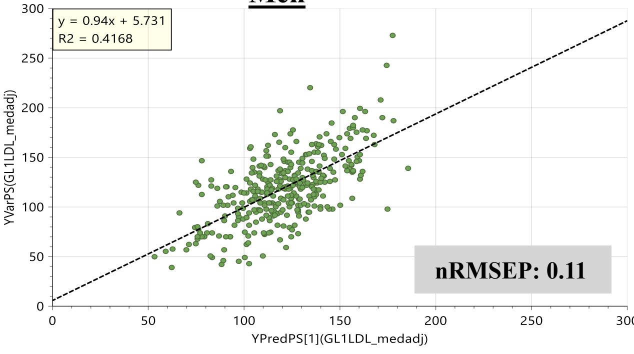

d) High density lipoprotein-cholesterol

Women

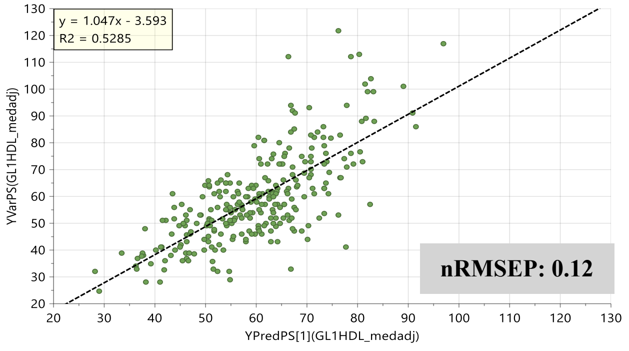

Men

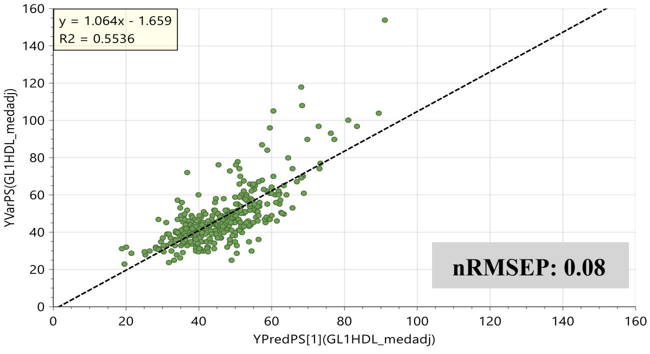

nRMSEP: normalized root mean square error of prediction; lower nRMSEP indicates higher prediction accuracy

Supplemental Table S3 a - d. Unstratified model results for metabolic pathways differentially associated with lipid measures by sex in the Coronary Artery Risk Development in Young Adults Study, 2005–2006 (N = 2,169)

*See excel file: Metabolites_3670261_SuppTable3_metabolic pathway analysis_linear regression models

<u>Regression Model Formulas:</u>

Model 1 (Basic Demographic Model): Clinical lipid measure = β_0 + β_1(Metabolite Peak) + β_2(Sex) + β_3(Sex × Metabolite Peak) + β_4(Batch) + β_5(Field Center) + β_6(Age) + β_7(Race) + β_8(Education) + β_9(Total Energy) + ε

Model 2 (Lifestyle Model): Clinical lipid measure = β_0 + β_1(Metabolite Peak) + β_2(Sex) + β_3(Sex × Metabolite Peak) + β_4(Batch) + β_5(Field Center) + β_6(Age) + β_7(Race) + β_8(Education) + β_9(Total Energy) + β_{10}(Smoking Status) + β_{11}(Alcohol Consumption) + β_{12}(Birth Control Use-women only) + β_{13}(Physical Activity) + ε

Model 3 (Clinical Model): Clinical lipid measure = β_0 + β_1(Metabolite Peak) + β_2(Sex) + β_3(Sex × Metabolite Peak) + β_4(Batch) + β_5(Field Center) + β_6(Age) + β_7(Race) + β_8(Education) + β_9(Total Energy) + β_{10}(eGFR) + β_{11}(Diabetes) + β_{12}(BMI) + β_{13}(Hypertension) + ε

Model 4 (Fully-Adjusted Model): Clinical lipid measure = β_0 + β_1(Metabolite Peak) + β_2(Sex) + β_3(Sex × Metabolite Peak) + β_4(Batch) + β_5(Field Center) + β_6(Age) + β_7(Race) + β_8(Education) + β_9(Total Energy) + β_{10}(Smoking Status) + β_{11}(Alcohol Consumption) + β_{12}(Birth Control Use-women only) + β_{13}(Physical Activity) + β_{14}(eGFR) + β_{15}(Diabetes) + β_{16}(BMI) + β_{17}(Hypertension) + ε

Model 5 (Sensitivity Model: Fully-Adjusted Model + Lipid-Lowering Medication): Clinical lipid measure = β_0 + β_1(Metabolite Peak) + β_2(Sex) + β_3(Sex × Metabolite Peak) + β_4(Batch) + β_5(Field Center) + β_6(Age) + β_7(Race) + β_8(Education) + β_9(Total Energy) + β_{10}(Smoking Status) + β_{11}(Alcohol Consumption) + β_{12}(Birth Control Use-women only) + β_{13}(Physical Activity) + β_{14}(eGFR) + β_{15}(Diabetes) + β_{16}(BMI) + β_{17}(Hypertension) + β_{18}(Lipid-Lowering Medication) + ε

Supplemental Figure S3. Unstratified model 1 (basic demographic model) [a] results for statistically significant metabolic pathways (FET < 0.05) differentially associated with at least one clinical lipid measure by sex in the Coronary Artery Risk Development in Young Adults Study, 2005–2006

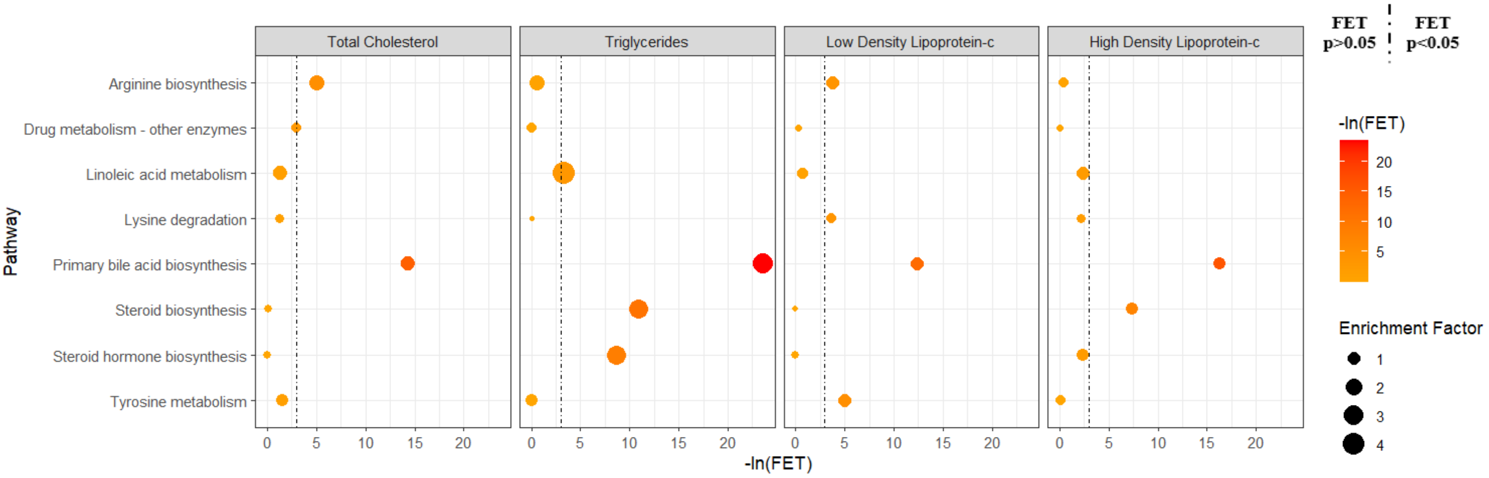

[a] Unstratified Model 1 (Basic Demographic Model): Clinical lipid measure = $\beta_0 + \beta_1$(Metabolite Peak) + β_2(Sex) + β_3(Sex × Metabolite Peak) + β_4(Batch) + β_5(Field Center) + β_6(Age) + β_7(Race) + β_8(Education) + β_9(Total Energy) + ε; Differential metabolic activity was determined by Pathway Enrichment Analysis using mass-to-charge ratio and ranked p-values from metabolite terms in linear regression models in Mummichog algorithm (v2) in MetaboAnalyst (v5) with human KEGG pathway mapping.[108]; FET p<0.05 tested whether there was greater metabolic activity in the pathway than expected by chance with darker red colors indicate greater significance [108]; Enrichment Factor represents the ratio of the number of metabolites found vs. expected in a pathway with higher ratios (larger circles) indicating greater pathway activity than expected by chance. [108]

FET, Fisher’s exact test; HDL-c, high density lipoprotein-cholesterol; LDL-c, low density lipoprotein-cholesterol; TC, total cholesterol; TG, triglycerides

Supplemental Figure S4. Unstratified model 2 (lifestyle-adjusted model) [a] results of significant metabolic pathways (FET < 0.05) differentially associated with at least one clinical lipid measure by sex in the Coronary Artery Risk Development in Young Adults Study, 2005–2006

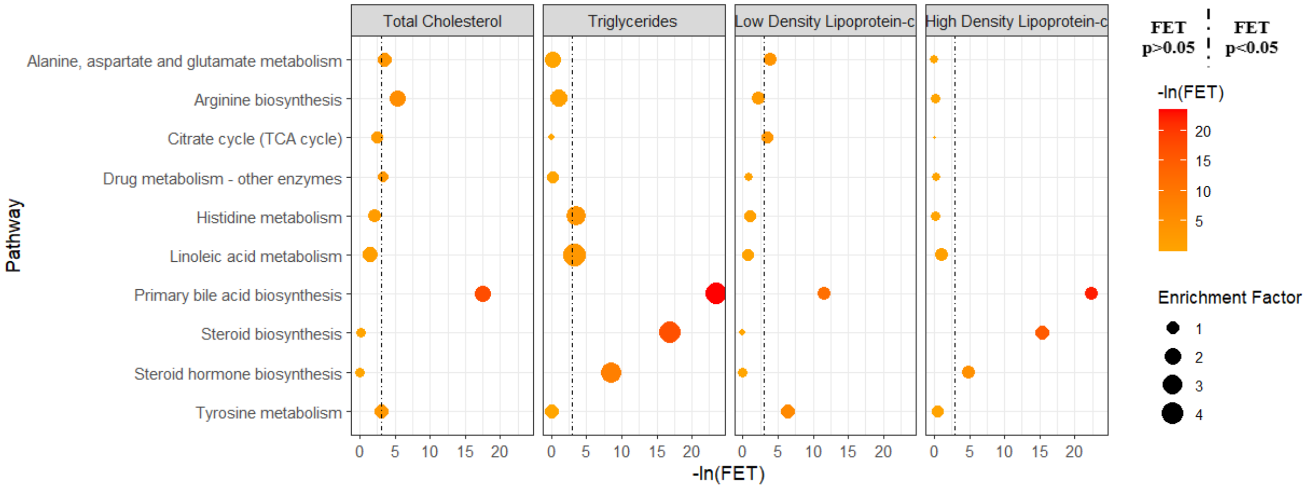

[a] Unstratified Model 2 (Lifestyle Model): Clinical lipid measure = $\beta_0 + \beta_1$(Metabolite Peak) + β_2(Sex) + β_3(Sex × Metabolite Peak) + β_4(Batch) + β_5(Field Center) + β_6(Age) + β_7(Race) + β_8(Education) + β_9(Total Energy) + β_{10}(Smoking Status) + β_{11}(Alcohol Consumption) + β_{12}(Birth Control Use-women only) + β_{13}(Physical Activity) + ε; Differential metabolic activity was determined by Pathway Enrichment Analysis using mass-to-charge ratio and ranked p-values from metabolite terms in linear regression models in Mummichog algorithm (v2) in MetaboAnalyst (v5) with human KEGG pathway mapping.[108]; FET p<0.05 tested whether there was greater metabolic activity in the pathway than expected by chance with darker red colors indicate greater significance [108]; Enrichment Factor represents the ratio of the number of metabolites found vs. expected in a pathway with higher ratios (larger circles) indicating greater pathway activity than expected by chance. [108]

FET, Fisher's exact test; HDL-c, high density lipoprotein-cholesterol; LDL-c, low density lipoprotein-cholesterol; TC, total cholesterol; TG, triglycerides

Supplemental Figure S5. Unstratified model 3 (clinical-adjusted model)[a] results of significant metabolic pathways (FET < 0.05) differentially associated with at least one clinical lipid measure by sex in the Coronary Artery Risk Development in Young Adults Study, 2005–2006

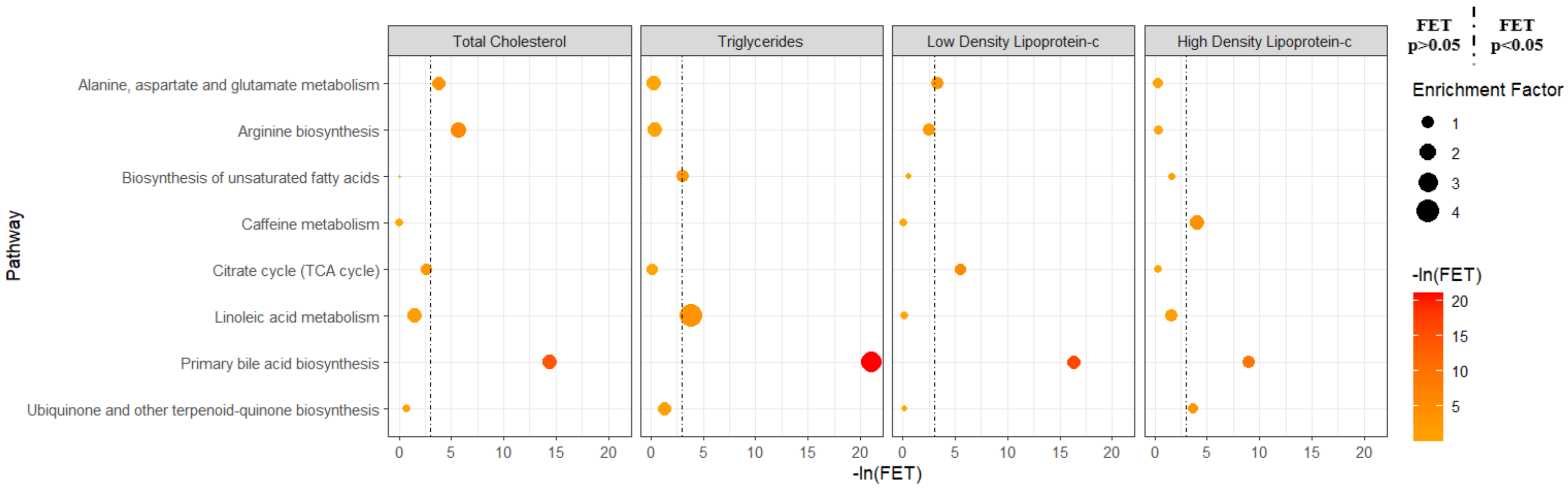

[a] Unstratified Model 3 (Clinical Model): Clinical lipid measure = $\beta_0 + \beta_1$(Metabolite Peak) + β_2(Sex) + β_3(Sex × Metabolite Peak) + β_4(Batch) + β_5(Field Center) + β_6(Age) + β_7(Race) + β_8(Education) + β_9(Total Energy) + β_{10}(eGFR) + β_{11}(Diabetes) + β_{12}(BMI) + β_{13}(Hypertension) + ε; Differential metabolic activity was determined by Pathway Enrichment Analysis using mass-to-charge ratio and ranked p-values from metabolite terms in linear regression models in Mummichog algorithm (v2) in MetaboAnalyst (v5) with human KEGG pathway mapping.[108]; FET p<0.05 tested whether there was greater metabolic activity in the pathway than expected by chance with darker red colors indicate greater significance [108]; Enrichment Factor represents the ratio of the number of metabolites found vs. expected in a pathway with higher ratios (larger circles) indicating greater pathway activity than expected by chance. [108]

FET, Fisher's exact test; HDL-c, high density lipoprotein-cholesterol; LDL-c, low density lipoprotein-cholesterol; TC, total cholesterol; TG, triglycerides

Supplemental Table S4 a - d. Stratified model results in men and women (by menopausal status) for metabolic pathways significant associated (FET < 0.05) with at least one clinical lipid measure by sex in the Coronary Artery Risk Development in Young Adults Study, 2005–2006

*See excel file: Metabolites_3670261_SuppTable4_metabolic pathway analysis_linear regression models_men and menopausal status

Regression Model Formulas:

Model 4 (Fully-Adjusted Model): Clinical lipid measure = $\beta_0 + \beta_1$(Metabolite Peak) + β_2(Sex) + β_3(Sex × Metabolite Peak) + β_4(Batch) + β_5(Field Center) + β_6(Age) + β_7(Race) + β_8(Education) + β_9(Total Energy) + β_{10}(Smoking Status) + β_{11}(Alcohol Consumption) + β_{12}(Birth Control Use-women only) + β_{13}(Physical Activity) + β_{14}(eGFR) + β_{15}(Diabetes) + β_{16}(BMI) + β_{17}(Hypertension) + ε

Supplemental Table S5 a – d. Unstratified and stratified OPLS-R metabolic pathway enrichment results of pathways significantly associated (VIP score > 1.5, FET < 0.05) with at least one clinical lipid measure in the Coronary Artery Risk Development in Young Adults Study, 2005–2006

*See excel file: Metabolites_3670261_SuppTable5_metabolic pathway analysis_OPLS-R_unstratified and stratified_fully adjusted models

Regression Model Formula:

Model 4 (Fully-Adjusted Model): Clinical lipid measure = β_0 + β_1(Metabolite Peak) + β_2(Sex) + β_3(Sex × Metabolite Peak) + β_4(Batch) + β_5(Field Center) + β_6(Age) + β_7(Race) + β_8(Education) + β_9(Total Energy) + β_{10}(Smoking Status) + β_{11}(Alcohol Consumption) + β_{12}(Birth Control Use-women only) + β_{13}(Physical Activity) + β_{14}(eGFR) + β_{15}(Diabetes) + β_{16}(BMI) + β_{17}(Hypertension) + ε

Supplemental Table S6. Unstratified fully-adjusted linear regression results of metabolite peak features associated with each clinical lipid measure in the Coronary Artery Risk Development in Young Adults Study, 2005–2006

*See excel file: Metabolites_3670261_SuppTable6_FDRvalues

Regression Model Formula:

Model 4 (Fully-Adjusted Model): Clinical lipid measure = β_0 + β_1(Metabolite Peak) + β_2(Sex) + β_3(Sex × Metabolite Peak) + β_4(Batch) + β_5(Field Center) + β_6(Age) + β_7(Race) + β_8(Education) + β_9(Total Energy) + β_{10}(Smoking Status) + β_{11}(Alcohol Consumption) + β_{12}(Birth Control Use-women only) + β_{13}(Physical Activity) + β_{14}(eGFR) + β_{15}(Diabetes) + β_{16}(BMI) + β_{17}(Hypertension) + ε

Supplemental Table S7 a – d. Unstratified and stratified OPLS-R results of metabolite peak features and VIP scores associated with each clinical lipid measure in the Coronary Artery Risk Development in Young Adults Study, 2005–2006

*See excel file: Metabolites_3670261_SuppTable7_VIPscores

OPLS-R Model Formula:

Model 4 (Fully-Adjusted Model): Clinical lipid measure = β_0 + β_1(Metabolite Peak) + β_2(Sex) + β_3(Sex × Metabolite Peak) + β_4(Batch) + β_5(Field Center) + β_6(Age) + β_7(Race) + β_8(Education) + β_9(Total Energy) + β_{10}(Smoking Status) + β_{11}(Alcohol Consumption) + β_{12}(Birth Control Use-women only) + β_{13}(Physical Activity) + β_{14}(eGFR) + β_{15}(Diabetes) + β_{16}(BMI) + β_{17}(Hypertension) + ε

Supplemental Table S8. Metabolic pathways identified from 7,488 metabolite peaks (excluding metabolites peaks with >80% missingness) associated with each clinical lipid measure with differential metabolic activity by sex in the Coronary Artery Risk Development in Young Adults Study, 2005–2006 (N = 2,169)

*See excel file: Metabolites_3670261_SuppTable8_exclude_missing

Model 4 (Fully-Adjusted Model): Clinical lipid measure = $\beta_0 + \beta_1$(Metabolite Peak) + β_2(Sex) + β_3(Sex × Metabolite Peak) + β_4(Batch) + β_5(Field Center) + β_6(Age) + β_7(Race) + β_8(Education) + β_9(Total Energy) + β_{10}(Smoking Status) + β_{11}(Alcohol Consumption) + β_{12}(Birth Control Use-women only) + β_{13}(Physical Activity) + β_{14}(eGFR) + β_{15}(Diabetes) + β_{16}(BMI) + β_{17}(Hypertension) + ε

Supplemental Figure S6. Principal components analysis of all study samples, study pools, NIST reference samples, and blanks using all 7522 metabolomics features used in the Coronary Artery Risk Development in Young Adults Study, 2005–2006

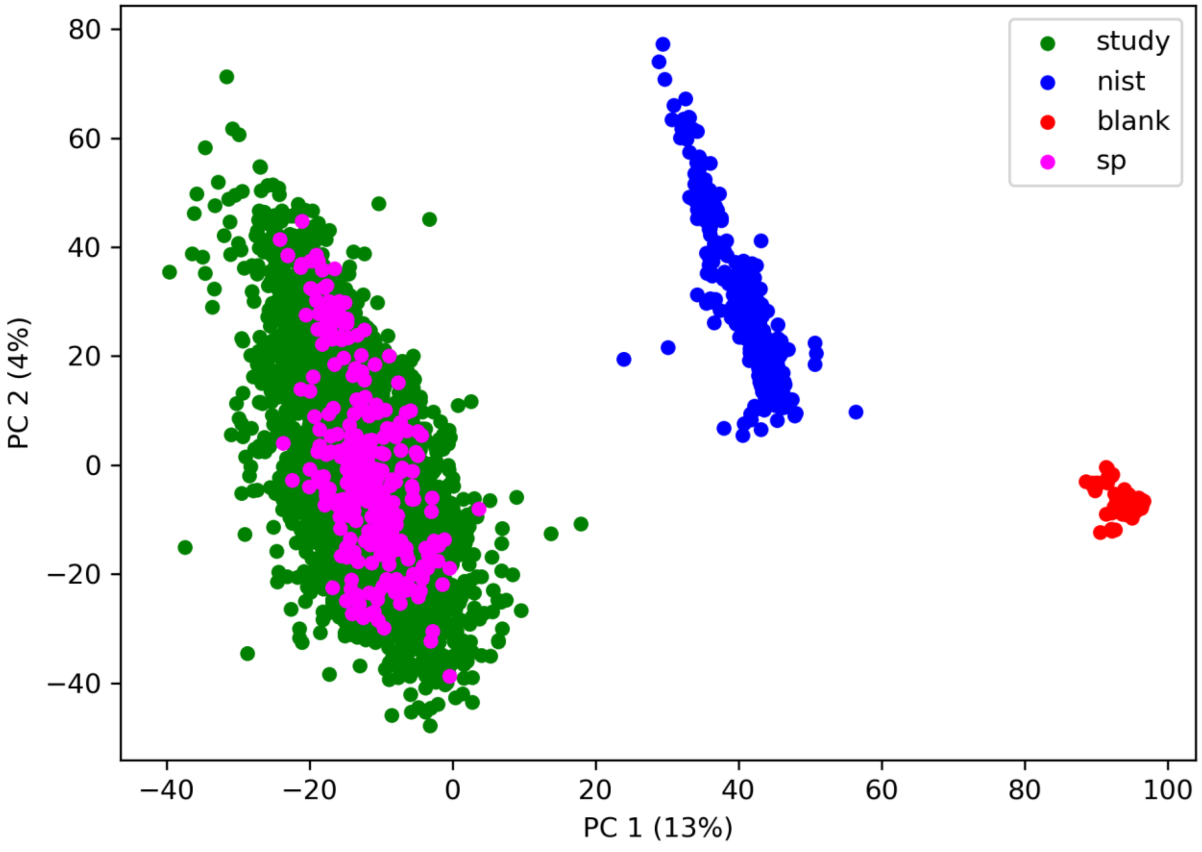

Data were scaled to unit variance; Study: refers to the individual participants included in the study; Blank: quality control samples; NIST: plasma reference material purchased from the National Institute of Standards and Technology (NIST) that represent pooled plasma samples taken from external participants outside of this study; SP: pooled sample made from all of the study samples that represent an "average" of the study samples

SUPPLEMENTAL METHODS

Supplemental methods have been published in sections of a dissertation thesis. [34]

Untargeted Metabolomics Profiling. CARDIA biospecimens were collected under standardized protocols for fasting blood collection and processing across clinic sites and were appropriate for metabolomics profiling. Participants were asked to fast ≥ 12 hours and to not smoke or exercise heavily 2 hours prior to the exam. Blood samples were collected using Fisher Scientific, #02-688-26 citrate vacutainer tubes, immediately put on ice for 30-40 mins, centrifuged at 3000 rpm for 20 minutes, and aliquoted and stored in Fisher Scientific, #02-681-343 citrate tubes at -70º C. The resulting plasma samples were then randomized into batch sizes of 80 samples and underwent one freeze-thaw to aliquot samples into new tubes at a volume of 50 µL. Plasma aliquots were then extracted using 400 µL of methanol containing 500 ng/mL of tryptophan-d5 as an internal standard. Samples were vortexed at 5000 revolutions per minute (rpm) for 2 minutes, centrifuged at 16,000 x g for 10 minutes at 4 ◦C, and 350 µL of the supernatant was transferred to new tubes and dried by speedvac overnight. To reconstitute samples, 100 µL of a 95:5 water-methanol was added to dried samples and vortexed for 10 minutes at 5000 rpm and then centrifuged at 16,000 x g at 4 ◦C for 10 minutes. Supernatants were then transferred to pre-labeled auto-sampler vials.

External Reference Material. National Institutes of Standards and Technology (NIST) pooled plasma samples (SRM 1950) were purchased from NIST, aliquoted into 50 µL aliquots, and stored at -80 °C. NIST aliquots were prepared exactly as the individual study samples and analyzed within and across batches (8 reference materials per 80 study samples) to evaluate intra- and inter-batch signal variation.

Batch Quality Control Study Pools. Quality control (QC) study pools were created by batch by mixing 10 µL of each of the 80 study samples in the batch and then re-aliquoted into volumes of 50 µL which were then prepared exactly as the individual study samples. Following data acquisition, statistical and

multivariate analysis were used to ensure that QC study pools were tightly clustered in Principle Component Analysis plots and were in the middle of the samples from which they were derived.

Untargeted ultra-high performance liquid chromatography–high-resolution mass spectrometry (UHPLC-MS) method. Plasma samples were analyzed by untargeted metabolomics which was conducted using Vanquish ultra high-performance liquid chromatography (UHPLC-MS) coupled with Q ExactiveTM HF-X Hybrid Quadrupole-OrbitrapTM Mass Spectrometer (Thermo Fisher Scientific, San Jose, CA) by the Metabolomics and Exposome Laboratory (Sumner, Director) at the University of North Carolina Nutrition Research Institute. Metabolites were separated using reversed-phase chromatography via a Waters Acquity HSST3 column 392,393. Mass spectrometry (MS) data were collected from a 70-1050 mass-to-charge ratio (m/z) range in positive mode. MS/MS data were acquired via the data-dependent acquisition scan to fragment the top 20 abundant precursor ions in each scan cycle. A C-mix solution comprised of 9 compounds with retention time spanning the chromatographic run period was used for pre- and post-analysis system suitability checks to evaluate signal intensity, retention time stability, and mass accuracy for each batch.

Data Processing. Plasma samples were analyzed in 42 analytical batches. Automated Data Analysis Pipeline (ADAP-BIG), a desktop software tool, was used to process the raw untargeted LC-MS/MS metabolomics data and extract compound information from the data.[31,109–111] The data processing conducts a sequence of steps, including peak detection, peak filtering, alignment, batch-effect correction, and normalization. Statistical and multivariate methods help ensure QC study pools are clustered and represent the average of the samples from which they were derived. This process produced a total of 7,522 metabolite peaks.

Missing Data. Missing data could occur in one of two forms: a peak missing from an entire batch or a peak missing from an individual sample within a batch. Due to the large number of samples, ADAP-BIG processes each batch separately and then aligns the batches one by one. When one batch is aligned with all the other batches that have been aligned, a peak will appear missing from the entire new batch if it

does not have a close-enough counterpart in the new batch. Missing abundance was expected due to one or a combination of factors: stringent alignment parameters, peak shape, low intensity, or co-elution with other compounds. We anticipated missing data was due to both missing at random and informatively missing mechanisms. However, we are unable to distinguish which missing data arose from which mechanism. Therefore, we used random forest imputation for our primary analysis, which has been shown to outperform other approaches to handling missing data in these scenarios.[29,30] Additionally, due to the metabolomics platform used, it is possible that certain metabolomic pathways were better preserved in fasted samples than others. We applied pareto scaling to peak data for Orthogonal Partial Least Squares – Regression (OPLS-R) as it weights metabolites with larger estimated effect sizes more heavily than those with smaller estimated effect sizes while still ensuring that these metabolites are not overly influential.[112] Finally, metabolite data were median-scaled and $\log_2$ transformed to improve normality.

Peak identification and annotation. All 7,522 peaks were searched against an in-house physical standard library (IPSL) or public databases (NIST, Human Metabolome Database) which contained retention time (RT, IPSL only), exact mass (MS), and fragmentation (MS/MS) data to match signals with compounds. Of the 7,522 metabolite peaks, 718 were identified/annotated by ADAP to 318 compounds using at least two orthogonal data (ontology level OL1, OL2a, OL2b, or PDA). As is common in untargeted metabolomics, not all metabolites can be annotated. For those that can be annotated, it should be noted that the current untargeted metabolomics platform used in this study cannot always differentiate isomer(s) of the reference standard peaks and therefore, multiple peaks can be annotated with the same compound name. Additionally, it is possible that multiple peaks are associated with different adducts for the same molecule. IPSL matches starting from the highest level of confidence include: 1) OL1 - identification based on matching with IPSL via retention time (RT) with RT error ≤0.5 minutes, exact mass with mass error <5 ppm, and tandem mass spectra similarity between the experimental fragmentation spectra and library spectra with spectra similarity ≥30, 2) OL2a - identification based on matching with IPSL via mass and RT, and 3) OL2b which denotes an annotation for the isomer or

derivatives of the compound listed, based on matching with IPSL via mass and MS/MS. Public database (PD) matches were assigned at one of two confidence levels. A PDA level denotes an annotation based on matching by mass and experimentally determined MS/MS. A PDD level denotes an annotation based on matching by mass only.
